# Ultrasound-assisted preparation of calcium malate and its absorption

**DOI:** 10.1371/journal.pone.0254583

**Published:** 2021-07-15

**Authors:** Wuren Ma, Yizhou Lv, Xuan Cao, Mengzhi Wang, Yunpeng Fan, Yuanyuan Shan

**Affiliations:** 1 College of Veterinary Medicine, Northwest A&F University, Yangling, Shaanxi, P R China; 2 College of Food Science & Engineering, Northwest A&F University, Yangling, Shaanxi, P R China; Universita degli Studi del Molise, ITALY

## Abstract

In this experiment, response surface methodology was used to study the preparation of malic acid calcium salt from bovine bones assisted by ultrasonication. The results showed that the optimum conditions for ultrasound-assisted preparation of calcium malate from bovine bone were as follows: solid-liquid ratio 1:15, solid-acid ratio 1:1.5, ultrasonic power 200 W, ultrasonic temperature 35°C, and ultrasonication time 17 min. The efficiency of calcium recovery was 66.16%, and the purity was 92.54%. After three ultrasonic treatments of 17 min each, the calcium malate conversion rate of bovine bone reached 95.73%. Animal experiments showed that feeding bovine bone-derived calcium malate significantly increased alkaline phosphatase (ALP) activity and bone calcium content, reduced tartrate-resistant acid phosphatase (TRAP) activity, and maintained the balance of serum calcium and phosphorus. These results indicated that the ultrasonic method effectively ionized calcium in bovine bone, which provides a reference point for the industrial production of calcium products with bovine bone as the raw material.

## 1. Introduction

Bovine bone is rich in minerals such as calcium, phosphorus and iron, which have a wide range of potential uses in foods and medicines [1, 2]. However, apart from ribs and bone cavities, other bone products cannot be utilized effectively, resulting in a waste of resources and environmental pollution [3, 4]. It is of great significance for the food industry to make full use of bone resources.

Currently reported methods for improving the solubility of bone calcium include acid hydrolysis, alkaline hydrolysis, enzymatic hydrolysis and microbial fermentation [5–7]. To some extent, these methods improve the dissolution rate of bone calcium, but acid and alkali hydrolysis can cause the irreversible denaturation of proteins in bone. Microorganism fermentation works well but, at 36.9%, the calcium conversion rate is low. In recent years, new technologies, such as a high voltage pulsed electric field [4, 8] and induction electric field-assisted technology [9], have also been used to effect bone calcium dissolution. Ultrasonic technology can theoretically destroy the combination of collagen and hydroxyapatite, accelerate the diffusion of solute, promote the dissolution of effective substances, and improve reaction rates [10]. The ultrasonic-assisted method has been widely used in the extraction and preparation of biologically active substances [11–13] and the transformation of organic calcium from eggshells and fish scale [14, 15], but it has seldom been reported in the transformation of organic calcium from livestock and poultry bones.

Calcium malate exemplifies a new generation of calcium sources, and it is an odorless white powder. Its absorption efficiency is 2.6 times that of ordinary calcium carbonate, and it exhibits little gastrointestinal stimulation [16]. It can be used as a calcium supplement for humans and animals and it is used in the pharmaceutical industry for diagnosis and treatment of osteoporosis [17, 18]. Calcium malate (chemical formula C_4_H_4_CaO_5_, molecular weight 172.15) is also used as a safe food additive, buffer and acidity regulator in the food industry. Calcium malate is also considered to be the most potentially marketable feed additive used in aquaculture [19, 20]. Therefore, converting hydroxyapatite from bovine bone into calcium malate, which can be easily absorbed by the human body, has broad potential for application. The purpose of this study was to explore new technology for preparing calcium malate from bovine bone by using ultrasound-assisted direct neutralization to improve the efficiency of calcium isolation, shorten the reaction time, and solve the environmental pollution problems caused by waste bovine bone.

## 2. Materials and methods

### 2.1. Materials and reagents

The long bones of bovines were purchased from the vegetable market of Northwest A&F University. NaOH, hydrochloric acid, nitric acid, calcium carbonate and EDTA-Na_2_ (analytically pure) were all purchased from Sinopharm Chemical Reagent Co., Ltd. The serum ALP kit, serum TRAP kit, serum calcium kit and phosphorus kit were all purchased from Nanjing Jiancheng Institute of Biological Engineering.

### 2.2. Animals

Thirty male rats weighing 90 g to 110 g (4 weeks old) were purchased from the Experimental Animal Center of the Air Force Medical University (Xian, China). Rats were housed at temperatures ranging from 20–24°C and with humidity levels ranging from 54–58%. Animal feed was made with the basic formula, and the calcium content was adjusted to 150 mg/100 g feed; free access to water was provided. All rats were adapted to the light/dark cycle for at least one week before the experiment. This study was carried out in strict accordance with the recommendations in the Guide for the Care and Use of Laboratory Animals of the National Institutes of Health. The experiments were performed with formal approval from the Committee on the Ethics of Animal Experiments of Northwest A&F University (No. 20190318).

### 2.3. Methods

#### 2.3.1. Ultrasonic processing

After washing, the long bones of bovines were broken into pieces of 10–20 cm, and then the bone pieces were cooked for 1 h at a pressure of 0.1 MPa and a temperature of 121°C. The boiled bone was placed in the oven and dried at 40°C for 4 h. After that, it was crushed by a multifunctional pulverizer and sieved by a 150 mesh sieve to make sure the uniformity of the bone particle size. Bone powder was weighed and placed in a beaker, and a certain proportion of malic acid solution was added to the ultrasonic cleaner (SB-5200DTD, 800 W, 40 kHz, purchased from Ningbo Xinzhi Biotechnology Co., Ltd.) for ultrasonic reaction according to preset parameters; these involved 3 s cycles of ultrasound and 5 s delays. After the reaction was completed, filtration was conducted to remove insoluble substances, and the filtrate was collected for the determination of ionic calcium content, concentrated under vacuum and dried.

#### 2.3.2. Determination of calcium ionization efficiency

The filtrate was taken to a constant volume of 50 mL, 5 mL of the solution was added to a 250 mL conical flask, and distilled water was added to dilute the filtrate to 50 mL. Sodium hydroxide was added to adjust the solution pH to a value between 11–12, and 2–3 drops of calcium ion indicator were added. EDTA solution was slowly added to the wine red solution. When the solution turned from wine red to blue and the color did not fade after 30 s, the titration endpoint was recorded. The amounts of EDTA and distilled water consumed were recorded as blank controls, and the amount of EDTA consumed was recorded (Shan et al, 2019). The level of dissolution of calcium ions was calculated according to the following formula:

$$dissolution\;level\;of\;calcium\;ions\;(\%)=[40\times \beta \times (V‐V_{0})\times C_{EDTA})/(\alpha \times m)\times 100\%$$ (1)

Where C_EDTA_ is the concentration of the EDTA-Na_2_ solution in mol/L; V is the EDTA-Na_2_ volume in the sample in L; V_0_ is the EDTA-Na_2_ volume in the blank control in L; β is the dilution factor; m is the mass of bone meal in g; and α is the percentage of calcium in bone meal.

#### 2.3.3. Determination of purity and conversion of calcium malate

The powdered mass of calcium malate was accurately weighed by the decrementation method [21]. The volume was fixed at 100 mL in a volumetric flask. A 20 mL solution was placed in a 250 mL conical flask, 20 mL distilled water was added, and then the pH was adjusted to 11–12 with a 1 mol/L NaOH solution. The sample solution with the EDTA standard was titrated carefully until the wine red color completely faded and the solution was pure blue for 30 s, and this was recorded as the end point of the titration. The volume V of the EDTA standard solution consumed was recorded. Additionally, using distilled water as the blank control, the number of milliliters of EDTA standard liquid consumed was recorded as V_0_. The purity of calcium malate was calculated as follows:

$$purity\;of\;calcium\;malate\;(\%)=[(V‐V_{0})\times C_{EDTA}\times V_{1}\times M]/(V_{2}\times M_{1})\times 100\%$$ (2)

Where V is the EDTA-Na_2_ volume in sample in L; V_0_ is the EDTA-Na_2_ volume in the blank control in L; C_EDTA_ is the concentration of EDTA-Na_2_ in mol/L; V_1_ is the total volume of calcium malate solution in L; V_2_ is the titrated volume of calcium malate in L; M is molar mass of calcium malate in g/mol; and M_1_ is mass of calcium malate powder in g.

The conversion rate of calcium malate was calculated as follows:

$$conversion\;rate\;of\;calcium\;malate\;(\%)=(m_{0}\times p)/m\times 100\%$$ (3)

Where m_0_ is the mass of calcium malate crude powder in g; p is the purity of calcium malate in %; and m is the mass of bone meal in g.

#### 2.3.4. Single factor design

1. The effect of ultrasonic power on the dissolution rate of bovine bone calcium
  The effect of ultrasonic power (0, 50, 150, 250, 350, and 450 W) on the dissolution rate of bovine bone ion calcium was investigated with a fixed feed liquid ratio (g:mL) of 1:15, feed acid ratio (mass ratio of bovine bone powder to malic acid, as below) of 1:1.5, ultrasonic temperature of 35°C, and ultrasonication time of 20 min.
2. The effect of ultrasonication time on the dissolution efficiency of bovine bone calcium
  The effect of ultrasonication time (0, 5, 10, 15, 20, and 25 min) on the dissolution efficiency of bovine bone calcium ions was investigated under the following conditions: fixed material acid ratio of 1:1.5, material liquid ratio of 1:15, ultrasonic power of 250 W, and temperature of 35°C.
3. The effect of ultrasonication temperature on the dissolution of bovine bone ion calcium
  The effects of different ultrasonic temperatures (25, 30, 35, 40, and 45°C) on the dissolution efficiency of bovine bone calcium ions were investigated under the following conditions: fixed material acid ratio of 1:1.5, material liquid ratio of 1:15, ultrasonic power of 250 W, and ultrasonication time of 20 min.
4. Effect of the ratio of feed to acid on the dissolution rate of bovine bone calcium
  The effect of the feed acid ratio (1:0, 1:0.5, 1:1.0, 1:1.5, 1:2.0, 1:2.5) on the dissolution rate of bovine bone calcium ions was investigated with a fixed feed liquid ratio of 1:15, ultrasonic power of 250 W, temperature of 35°C, and ultrasonication time of 20 min.
5. The effect of the ratio of feed to liquid on the dissolution rate of bovine bone calcium
  The effect of the ratio of feed to liquid (1:5, 1:10, 1:15, 1:20, and 1:25) on the dissolution rate of bovine bone calcium ion was investigated with a fixed material acid ratio of 1:1.5, temperature of 35°C, ultrasonication time of 20 min, and ultrasonic power of 250 W.

#### 2.3.5. Response surface design

Based on a single factor tests run according to Box-Behnken test design requirements [22], ultrasonication time, ultrasonic power and material/acid ratio were taken as the study variables, and the conversion efficiency of calcium malate was the response value. Three factors and three levels of response surface analysis were designed by using Design Expert 8.0 software. The ultrasonication times were set as 16, 20, and 24 min, the feed-acid ratios were set as 1.0, 1.5, and 2, and the ultrasonic power levels were set as 200, 250, and 300 W. There were 17 experimental points in the experiment, 12 of which were analysis factors and 5 of which were zero points. The zero point experiment was carried out 5 times, and the experiment was carried out randomly to determine the estimated error and repeated 3 times.

#### 2.3.6. Effect of ultrasonic treatment on the dissolution of ionic calcium

Through the combination of the regression model and the actual production, the optimal process conditions for the ultrasonic preparation of calcium malate from bovine bone were determined. Using these conditions, 5 parallel experiments were carried out for verification. Moreover, for the experimental group that was not subjected to ultrasonic treatment, the reaction was run for 60 min for comparison.

#### 2.3.7. Effect of ultrasound cycles on the level of free calcium extracted from bovine bone

On the basis of the optimized technological parameters, a process for dissolving all bovine bone calcium was explored. In other words, after the bone powder, water and malic acid were mixed evenly in the optimal ratio, ultrasonic treatment was carried out, and supernatant and bone residue were obtained by centrifugation to determine the content of free calcium. The bone residue obtained by centrifugal separation was dried by colloidal grinding, and the above steps were repeated twice.

### 2.4. Animal experiments

#### 2.4.1. Animal grouping and dose setting

Rats were randomly divided into 3 groups according to their weight, with 10 animals in each group. Only a single dose level was used for the experimental group (EG) in the study, and the dose was determined to be 30 times the recommended intake of calcium supplements (calcium 420 mg/d) (calcium 210 mg/kg∙BW on a 60 kg scale). Calcium intake was the same in each group, and a low-calcium negative control group (NCG) and a calcium gluconate positive control group (PCG) were established. The rats were weighed each week and blood was collected. First, the rat tail was fixed and exposed, and the hair of the tail was cut off and disinfected. Then, the rat tail was soaked in warm water at approximately 50°C for several minutes to dilate the blood vessel in the tail. Finally, the tail was dried, and the tail vein was cut to collect the blood. Blood was allowed to clot at 4°C for 3 h, and the serum was separated and collected. Rats were euthanized by inhalation of pure carbon dioxide, and the femur of the left leg was removed immediately. Muscle tissue and connective tissue were wiped with gauze, and blood was washed with normal saline. Finally, the serum and femur of the left leg were collected to measure the contents of ALP, TRAP, calcium and phosphorus in the serum and the content of calcium in the femur using a kit and EDTA method described in GB/T 5009.92–2003.

#### 2.4.2. Method for determination of apparent absorption of calcium

Rats in each group were fed different calcium supplements continuously for 1 week, 5 weeks, and 10 weeks after 3 d of calcium metabolism experiments. The collected mouse feces were labeled in groups, dried and weighed in an oven at 70°C and ground into powder for digestion and treatment; the calcium content was then determined, and the apparent absorption rate of calcium (%) was calculated [23].


apparentabsorptionrateofcalcium(%)=(M1‐M2)/M1×100% (4)

Where M1 is the level of calcium intake in mg and M2 is the weight of calcium in feces in mg.

### 2.5. Data analysis

The test data are expressed as the mean ± standard deviation. Microsoft Office Excel 2013 and Design Expert 8.0.5b were used for processing, and Duncan’s multiple comparison test was used to test the significance of differences among various treatments (P < 0.05).

## 3. Results

### 3.1. Effects of ultrasonic power, ultrasonication time and ultrasonic temperature on the ionization efficiency of bovine bone calcium

**Fig 1:**
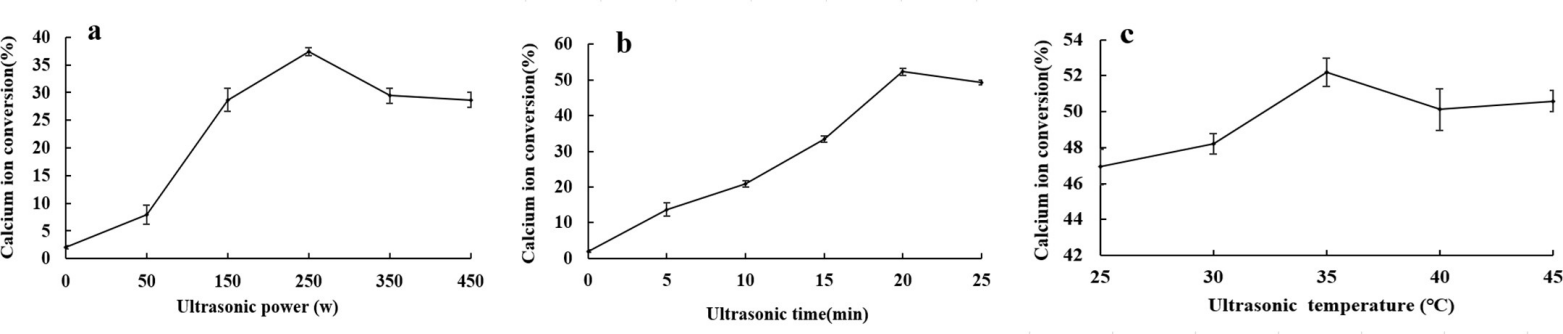
Effect of ultrasonic power, ultrasonic time and ultrasonic temperature on the free calcium extraction rate from bovine bone.

As shown in Fig 1A, the conversion level of ionic calcium increased significantly with increasing ultrasonic power in the range 0–350 W, and above 250 W, the rate of increase for ionic calcium level tended to diminish. Above 300 W, the conversion level of bovine bone calcium decreased.

It can be concluded from the data in Fig 1B that the level of conversion of ionic calcium increased with increasing ultrasonication time within the range 0–20 min, and the level of ionic calcium conversion increased slowly or even decreased beyond 20 min. Therefore, the ultrasonication time was set as approximately 18–22 min for the subsequent response surface optimization test, based on production practice and cost.

Fig 1C shows that the level of ion calcium conversion increased with increasing ultrasonic temperature within the range 0–40°C, and when the ultrasonic temperature was 40°C, the level of bone calcium ionization was significantly higher than those obtained at other temperatures. However, after the ultrasonic temperature exceeded 40°C, the efficiency of conversion of ionic calcium decreased with increasing ultrasonic temperature. Therefore, the range of ultrasonic reaction temperatures was set at 35–45°C for subsequent response surface optimization tests.

### 3.2. Effects of feed acid ratio and feed liquid ratio on the conversion of bovine bone source calcium malate

**Fig 2:**
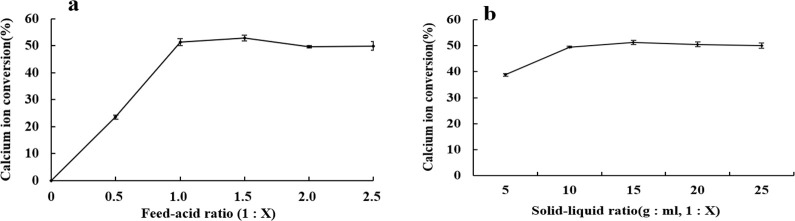
Effect of acid and liquid addition on the efficiency of free calcium extraction from bovine bone.

Fig 2A shows that the level of conversion of ionic calcium increased with increasing material acid ratio. When the ratio of bone meal to malic acid was increased to 1:1.5, the ionization efficiency of calcium increased significantly, and after this interval the increase in the level of ionic calcium conversion tended to moderate. Therefore, an acid ratio (mass ratio of bone powder to malic acid) of 1:1.5 was selected as the optimal condition.

Fig 2B shows that when the ratio of feed to liquid was below 1:15 (g: mL), the conversion of ionic calcium increased significantly with increasing liquid content. When the ratio of feed to liquid was 1:15, the calcium ionization efficiency reached the maximum value of 51.3%, and a further increase in the ratio had no significant effect on the calcium ionization level. After considering actual production costs, the 1:15 ratio of material to liquid was selected for the subsequent response surface optimization test.

### 3.3. Response surface analysis

#### 3.3.1. Establishment and analysis of the response surface regression model

Based on the results of single-factor experiments, the feed-acid ratio (1:1.5) and solid-liquid ratio (1:15) were fixed, the ultrasonic power (X_1_), ultrasonic temperature (X_2_) and ultrasonication time (X_3_) were used as test factors, the calcium ionization efficiency was used as the response value, and the response surface methodology (RSM) was used to optimize the preparation process for isolating malic acid calcium from bovine bone sources. The results are shown in Table 1. Multiple regression analysis was performed on the data by using Design-Expert 8.0 software. A quadratic regression model was established between the conversion efficiency of calcium malate (Y) and ultrasonication time (X_1_), acid-material ratio (X_2_) and ultrasonic power (X_3_), as shown in the following formula:

$$Y=51.53+1.63X_{1}+6.93X_{2}-1.97X_{3}-0.38X_{1}X_{2}+0.66X_{1}X_{3}-3.19X_{2}X_{3}-2.04X_{1}{}^{2}+0.57X_{2}{}^{2}+1.15X_{3}{}^{2}$$

**Table 1:** Optimization of response surface results.

Number	X_1_	X_2_	X_3_	Y
	Time (min)	Feed-acid ratio	Power (w)	Ca^2+^ conversion(%)
1	1	0	1	50.65
2	0	1	1	55.73
3	-1	0	-1	51.94
4	0	0	0	52.43
5	0	-1	-1	44.39
6	-1	0	1	46.90
7	0	-1	1	46.62
8	-1	1	0	54.50
9	0	0	0	51.14
10	0	0	0	50.60
11	1	0	-1	53.05
12	0	1	-1	66.26
13	-1	-1	0	41.53
14	0	0	0	52.82
15	1	-1	0	46.38
16	0	0	0	50.68
17	1	1	0	57.84

To further analyze the degree of fit of the regression equation obtained, ANOVA was conducted on the model, and the results are shown in Table 2. The results showed that the P value of the fitted equation was <0.0001, which indicates that the obtained equation is significant at the 99% level. The misfit term (0.1036) of the equation was not significant, indicating that the equation was well fitted, and the regression equation accurately analyzed and predicted the relationships between various factors and the calcium conversion efficiency. The results of the P value test showed that the p-values of the primary term X_1_ and interaction terms X_1_X_2_ and X_1_X_3_ of the model were less than 0.01, which represented an important contribution to the model. Therefore, the P values of the primary terms X_2_ and X_3_ and interaction terms X_2_X_3_ were less than 0.05, indicating that they had a significant impact on the transformation of organic calcium in bovine bone. According to the F test, the importance of factors affecting the efficiency of organic calcium conversion from bovine bone decreased in the order X_2_ (acid-material ratio) > X_3_ (ultrasonic power) > X_1_ (ultrasonic time).

**Table 2:** ANOVA for response surface quadratic model analysis of variance.

Source	Sum of Squares	df	Mean Square	F Value	p-value Prob > F	Significant
Model	502.19	9	55.8	35.25	< 0.0001	***
X_1_	21.31	1	21.31	13.46	0.008	**
X_2_	383.69	1	383.69	242.36	< 0.0001	***
X_3_	30.93	1	30.93	19.54	0.0031	**
X_1_X_2_	0.57	1	0.57	0.36	0.567	
X_1_X_3_	1.74	1	1.74	1.1	0.3296	
X_2_X_3_	40.65	1	40.65	25.67	0.0015	**
X_1_^2^	17.58	1	17.58	11.11	0.0125	*
X_2_^2^	1.37	1	1.37	0.86	0.3834	
X_3_^2^	5.54	1	5.54	3.5	0.1036	
Residual	11.08	7	1.58			
Lack of Fit	6.85	3	2.28	2.16	0.2353	◆
Pure Error	4.23	4	1.06			
Cor Total	513.27	16				

Notes: *, ** and *** represent P<0.05, P<0.01, and P<0.0001, respectively; ◆ represents “not significant”.

#### 3.3.2. Optimization of the preparation conditions for calcium extraction efficiency

**Fig 3:**
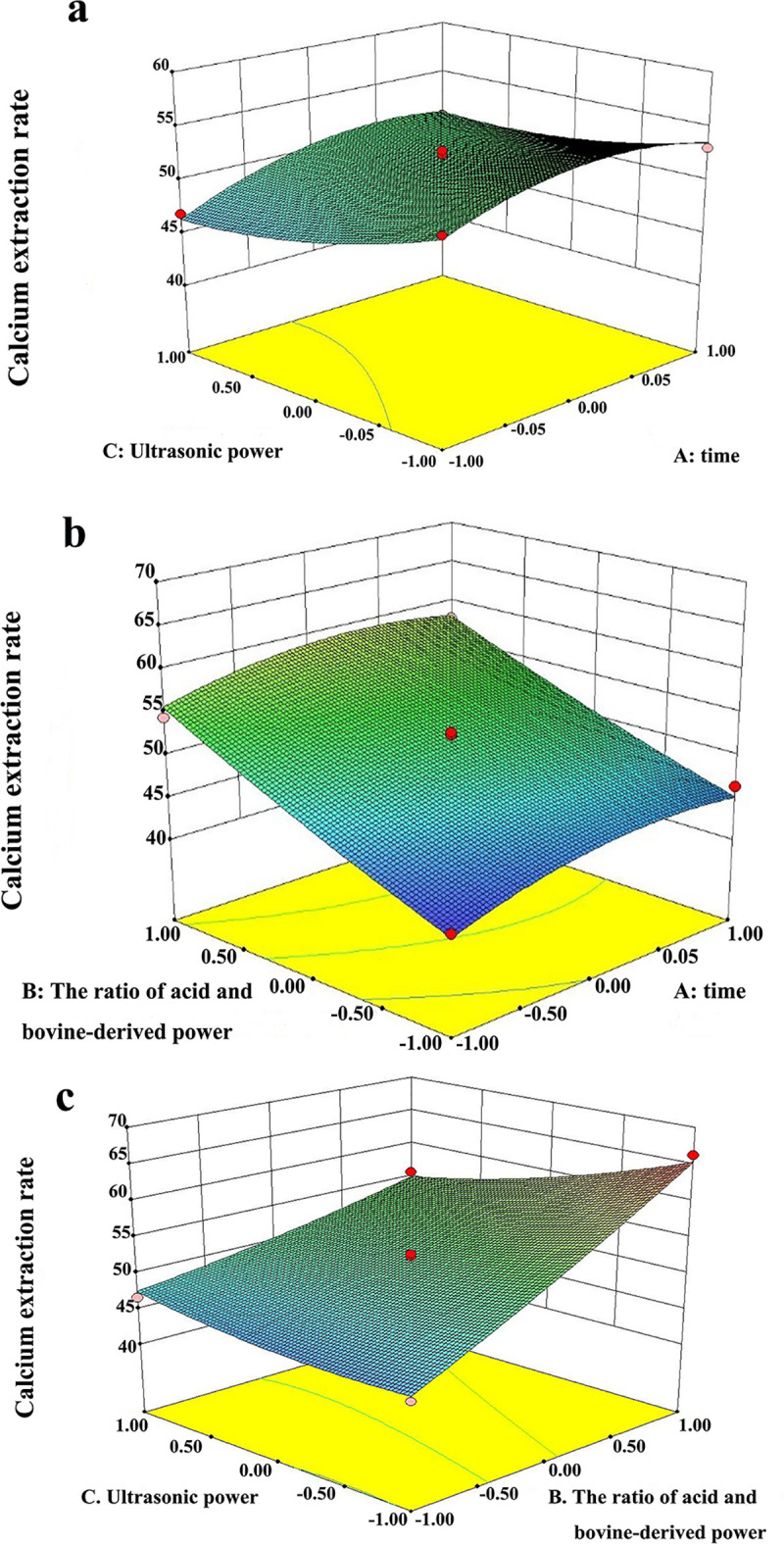
Response surface of calcium extraction rate. (a): The 3-D response surface plot for varying times and ultrasonic powers; (b): The 3-D response surface plot for varying times and the ratio of acid and bovine-derived powder; (c): The 3-D response surface plot for varying ratios of acid and bovine-derived powder and ultrasonic power.

The three-dimensional (3-D) surface images for the independent variables and dependent variables are presented in Fig 3. The center of the smallest ellipse in the contour diagram lies within the scope of three levels. This indicated that the maximum response value existed in the ranges of three levels. The maximum calcium extraction rate presumed by the regression model was 65.37%. At this time, the optimized conditions for preparation of bovine bone calcium malate are: ultrasonic power of 200 W, ultrasonication time of 16.6 min, reaction temperature of 35°C, ratio of feed to acid of 1:1.5 and ratio of feed to liquid of 1:15.

#### 3.3.3. Verification of optimal conditions

According to the predictions of the regression model and production practice, the ultrasonic power was 200 W, the reaction temperature was 35°C, the ultrasonication time was 17.0 min, the material acid ratio was 1:1.5, and the material liquid ratio was 1:15. Table 3 shows that with this optimal ultrasound-assisted process, the conversion efficiency of bovine bone source calcium malate reached 66.31%, which is close to the 65.37% level predicted theoretically; the difference is only 1.44%, which confirms the effectiveness of the model. Additionally, the purity of the calcium malate was 92.54%. The conversion efficiency for calcium malate in the 17 minute ultrasonic-assisted reaction was significantly higher than that of the 60 min conventional reaction, indicating that the ultrasonic-assisted reaction exhibited obvious advantages in promoting the speed and efficiency of organic calcium conversion in bovine bone.

**Table 3:** The yield of calcium malate for treatment with different methods.

Groups	Ultrasonic	No ultrasonic	Stirring	Cooking
Calcium malate conversion (%)	66.31±1.98	0.98±0.10	10.88±0.72	15.73±0.59
Calcium malate purity (%)	92.54±1.87	91.67±2.04	91.38±0.89	90.35±2.35

### 3.4. Effect of ultrasound cycles on the efficiency of free calcium extraction from bovine bone

After only one ultrasound treatment, 66.2% of the calcium in the bone was extracted. After a second ultrasonic treatment, 25.3% of the ionic calcium was also dissolved, and the total calcium ionization efficiency reached 91.5%. After three cycles of ultrasonic treatment of bovine bone meal, the conversion efficiency of soluble calcium reached 95.7%, and most of the calcium in bovine bone was converted to ionic calcium (Table 4).

**Table 4:** Effect of ultrasound cycles on the efficiency of free calcium extraction from bovine bone.

Times	Ca^2+^ in supernatant	Ca^2+^ in bone slag	Ca^2+^ conversion (%)
1	1455.52±8.20	744.45±6.77	66.16±3.08
2	188.60±3.23	556.32±7.02	25.27±1.32
3	24.02 ±1.34	532.30±10.63	4.30±0.69
Total			95.73± 4.76

Note: The calcium content of bovine bone meal without ultrasonic treatment was 220 mg/g.

### 3.5. Evaluation of the calcium supplementation effect for bovine bone calcium malate

**Fig 4:**
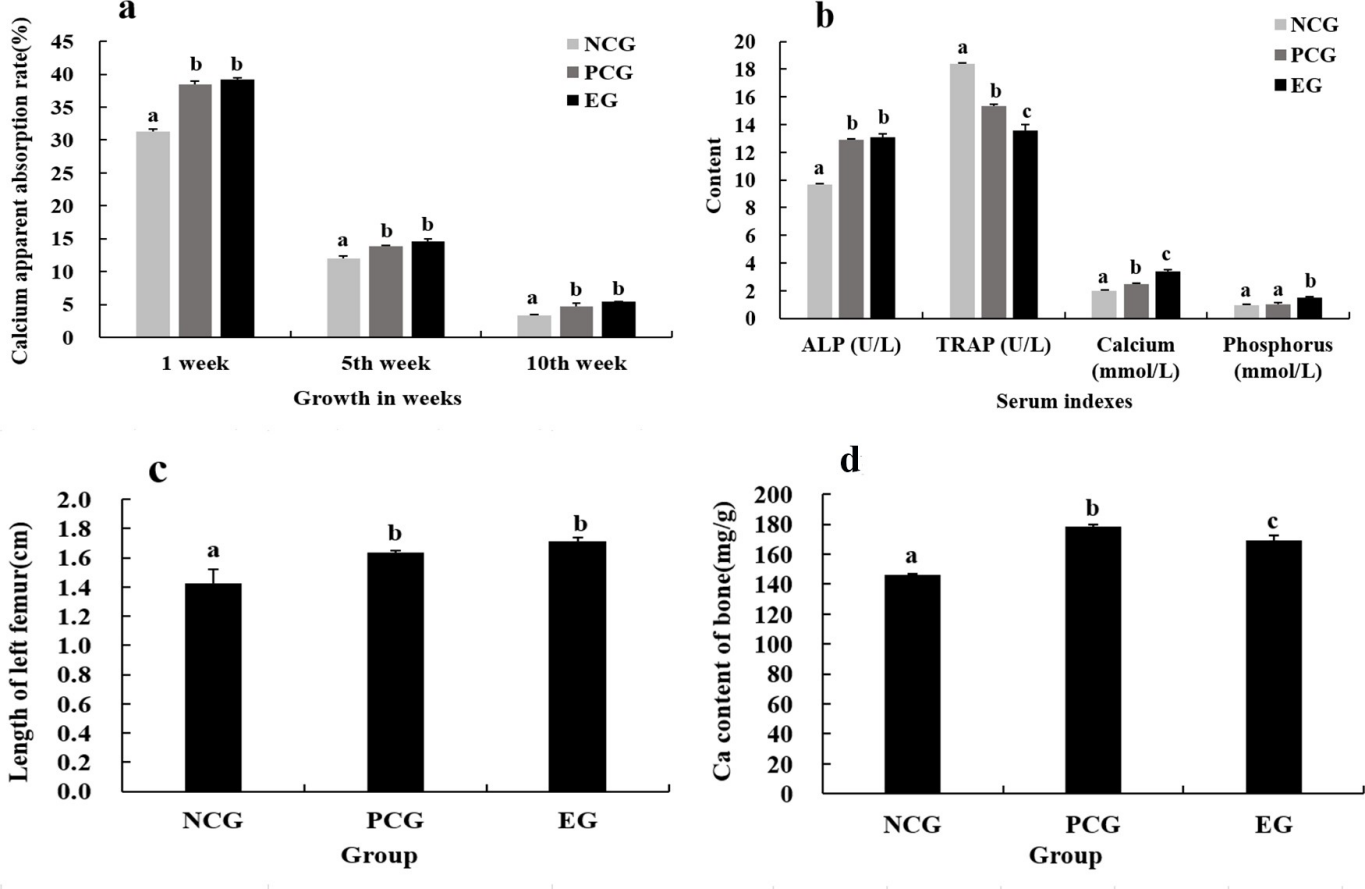
Evaluation of the effects of calcium supplementation with bovine bone calcium malate. (a) effect of different calcium sources on apparent absorption of calcium in rats; (b) effects of different calcium sources on serum indexes in rats; (c) & (d) effects of different calcium sources on femoral length and bone calcium levels in rats. Values for the same component were significantly different (p<0.05).

According to animal experiments (Fig 4A), the apparent absorption efficiencies for PCG and EG were significantly higher than that of NCG after calcium supplementation for one week, five weeks and ten weeks (P<0.05). However, there was no significant difference in apparent absorption between EG and PCG (P>0.05). The results indicated that the efficiency of absorption of calcium malate from bovine bone was similar to that of calcium gluconate.

Fig 4B shows that the ALP contents of PCG and EG were significantly different from that of NCG (P<0.05); the TRAP content of EG was significantly lower than those of NCG and PCG (P<0.05); the serum calcium contents of PCG and EG were significantly higher than that of NCG (P<0.05); and the serum phosphorus content of EG was significantly higher than those of NCG and PCG (P<0.05), indicating that feeding of bovine bone calcium malate for 70 d significantly improved ALP activity, reduced TRAP activity and maintained the balance of serum calcium and phosphorus.

In Fig 4C & 4D, the femoral lengths of the PCG and EG were significantly higher than that of the NCG (P<0.05). The bone calcium contents in the PCG and EG were significantly higher than that in the NCG (P<0.05). These results indicated that bovine bone calcium malate increased the length of the femur and the content of bone calcium.

## 4. Discussion

Bovine bones are rich in calcium and phosphorus. Most of the calcium in bovine bones is in the form of hydroxyl phosphate lime, and it is bound to collagen. In the process of preparing calcium malate from bovine bone, the use of ultrasonic mechanical vibration and cavitation broke up the bone collagen and hydroxyphosphate lime, and bone calcium was dissolved. In this experiment, with the increase of ultrasonic power, time, and temperature, the cavitation and mechanical vibration enhancement of ultrasonic helped the diffusion of malic acid molecules, thereby promoting the reaction of malic acid with the hydroxyapatite in the bovine bone. In addition, the interaction of ultrasound and the material liquid produces a large amount of foam. After the foam was broken, a local instantaneous pressure was generated, which promotes the fracture of bone collagen and was also beneficial to the dissolution of calcium from bovine bone. This process not only increases the contact area between bone meal and malic acid, but also greatly increases the chemical rate of the direct neutralization reaction between calcium ions and malic acid [24–26].

Ultrasonic treatment can promote contact between materials and solvents and speed up reactions. Many studies have proven that too much power, excessive time, extreme high temperature, and high material acid ratio and low material liquid ratio accelerate the decomposition of the generated material or change the physical properties of the product, which is not conducive to the reaction [27, 28]. This test has also confirmed this point. As shown in Fig 1A, with powers above 250 W, the increase in the calcium ion conversion efficiency tended to level. However, the conversion efficiency of bovine bone calcium decreased after 300 W, which may be related to degradation or property changes of calcium malate caused by treatment with ultrasound irradiation of excessive intensity [29]. As shown in Fig 1B, after 20 min, the ultrasonic wave diffused from the surface of bovine bone particles to the interiors of bovine bone particles, the generated calcium malate decomposed faster or its physical properties changed; this led to flocculation in the ultrasonic process and precipitation to different extents [27], which impeded the calcium malate conversion from bovine bone source. In Fig 1C, after the ultrasonic temperature exceeded 40°C, the efficiency of ionic calcium conversion decreased with continuing increases in ultrasonic temperature; this may be due to the high temperature, which reduces the solubility of calcium malate crystals and leads to a decrease in the content of calcium measured in solution [5]. In Fig 2A, when the ratio of feed to acid is higher than 1:1.5, the conversion rate of ionized calcium decreases. This may be due to the decomposition of calcium malate or changes in properties when the acid is too much. In Fig 2B, when the ratio of material to liquid was lower than 1:15, the conversion rate of ionized calcium decreases. The reason may be related to the solubility of calcium malate in water. Part of the precipitate calcium malate was filtered, resulting in a decrease in calcium content.

The response surface method established the multiple quadratic regression polynomial model equation according to the interactions between experimental factors and indicators, and this is more reliable than the orthogonal experimental optimization method [30, 31]. The results of this experiment showed that the optimal conditions obtained by response surface analysis predicted that the efficiency of calcium malate conversion from a bovine bone source would be 65.37%, which was close to the actual value of 66.31%. Additionally, the purity of the calcium malate was 92.54% under the optimum process conditions, which indicated that the process conditions were reasonable and feasible. The conversion efficiency for calcium malate after a 17 min ultrasonic-assisted reaction was significantly higher than that of the 60 min conventional reaction, indicating that the ultrasonic-assisted reaction had obvious advantages in promoting the speed and efficiency of calcium malate conversion in bovine bone.

The absorption level of calcium is often used as an important indicator of the quality of calcium supplements [32]. The results of animal experiments (Fig 4A) showed that the absorption rate of bovine bone calcium malate was significantly higher than that of the distilled water negative control group, which was equivalent to the absorption effect of commercially available calcium gluconate.

ALP is mainly derived from bone tissue and liver. When liver function is normal, the total ALP level can represent changes in bone metabolism (mainly osteogenesis) [33]. The animals used in this experiment were healthy rats, which excludes abnormal liver function. Therefore, the ALP value measured in serum directly reflects osteogenesis in experimental rats. Bone TRAP is released by osteoclasts and plays an important role in bone dissolution during bone resorption. When bone resorption is active, enzyme activity in the blood is significantly increased [34]. Serum calcium and phosphorus concentrations reflected bone mineral metabolism, and bone calcium content reflected calcium deposition. The results of the calcium supplementation effect of calcium malate prepared from eggshells by PEF [8] were similar to this study. These results showed (Fig 4B–4D) that a 70 d regimen of calcium malate significantly improved ALP activity, reduced TRAP activity, maintained the balance of serum calcium and phosphorus, and increased femoral length and bone calcium content.

## 5. Conclusion

The optimal process conditions for the isolation of malic acid calcium from bone sources are as follows: a material liquid ratio of 1:15 (g/mL), acid ratio of 1:1.5, ultrasonic power of 200 W, ultrasonication time of 17 min and ultrasonic temperature of 35°C. Under these conditions, the calcium conversion efficiency was 66.16%, and the purity was 92.54%. Animal experiments showed that feeding bovine bone-derived calcium malate (210 mg/kg∙BW) for 70 d significantly increased ALP activity and bone calcium content, reduced TRAP activity, and maintained the balance of serum calcium and phosphorus. The above results indicated that the ultrasonic-assisted method is an effective method for ionizing calcium in bovine bone, which provides a reference point for the industrial production of calcium products from bovine bone.

## Supporting information

S1 Data (XLSX)

S2 Data (XLS)
